# Clinical Management Update of Oral Leukoplakia: A Review From the American Head and Neck Society Cancer Prevention Service

**DOI:** 10.1002/hed.28013

**Published:** 2024-11-25

**Authors:** James C. Gates, Marianne Abouyared, Yelizaveta Shnayder, D. Gregory Farwell, Andrew Day, Faizan Alawi, Michael Moore, Andrew J. Holcomb, Andrew Birkeland, Joel Epstein

**Affiliations:** ^1^ Department of Oral and Maxillofacial Surgery Hospital of the University of Pennsylvania Philadelphia Pennsylvania USA; ^2^ Department of Otolaryngology—Head and Neck Surgery UC Davis Health Sacramento California USA; ^3^ Department of Otolaryngology—Head and Neck Surgery University of Kansas School of Medicine Kansas City Kansas USA; ^4^ Department of Otorhinolaryngology—Head and Neck Surgery Hospital of the University of Pennsylvania and Perelman School of Medicine Philadelphia Pennsylvania USA; ^5^ Department of Otolaryngology UT Southwestern Medical Center Dallas Texas USA; ^6^ Department of Oral and Maxillofacial Pathology, Penn Dental Medicine and Department of Cutaneous Biology, Perlman School of Medicine University of Pennsylvania Philadelphia Pennsylvania USA; ^7^ Department of Otolaryngology‐ Head and Neck Surgery Indiana University School of Medicine Indianapolis Indiana USA; ^8^ Department of Head & Neck Surgical Oncology Estabrook Cancer Center, Nebraska Methodist Hospital Omaha Nebraska USA; ^9^ Department of Otolaryngology‐ Head and Neck Surgery UC Davis Health Sacramento California USA; ^10^ Department of Surgery Cedars‐Sinai Health System Los Angeles California USA; ^11^ City of Hope Comprehensive Cancer Center Duarte California USA

**Keywords:** dysplasia, oral cancer, oral carcinogenesis, oral leukoplakia, oral premalignant disease

## Abstract

**Background:**

Oral potentially malignant disorders (OPMDs) occur in up to 4%–5% of the population, of which oral leukoplakia (OL) is the most common subtype. Predicting high‐risk OL remains a challenge. Early diagnosis and effective treatment are thought to be of paramount importance to improve outcomes.

**Methods:**

We searched PubMed and Clinicaltrials.gov data for updates in the clinical management of OL from 2015 to current.

**Results:**

Recent publication of large cohorts of patients with OL aids in counseling patients regarding risk of malignant transformation. Management for OL includes surveillance, excision, and laser surgery, as well as local and systemic approaches to chemoprevention. Several new entities show promise regarding candidate biomarkers, chemoprevention agents, and diagnostic adjuncts, though all require further validation.

**Conclusion:**

This update serves to further inform clinical management of OL and provide impetus for future investigations.

**Trial Registration:**

NCT00099021, NCT00951379, NCT05727761, NCT05727761

## 1 Introduction

The World Health Organization (WHO) maintains the classification of diseases that constitute “oral potentially malignant disorders” (OPMDs) [1]. The worldwide prevalence of OPMDs is 4.5%, and the most common type is oral leukoplakia (OL), which has an estimated prevalence of 1.39% worldwide and up to 9.10% in specific populations [2, 3, 4]. A recent meta‐analysis reported an overall malignant transformation (MT) rate for OL of 9.5% [5]. Population‐level data are predominately derived from studies outside the United States (US), so there is further uncertainty about prevalence of OL and OPMDs more broadly within the United States.

The epidemiology and risk factors of OL are well documented. Behavioral determinants, including tobacco, alcohol, and areca nut/betel quid use, are the most frequent causative agents in OL of all subtypes [5, 6, 7, 8]. Less is known regarding the risk of marijuana smoking and the development of OL. However, Gallagher et al. [9] highlight an association between cannabis use disorder and the development of head and neck cancer in adults. Other nonmodifiable risk factors for development of OL include advanced age, [7] immunosuppression, [7] and hereditary conditions such as dyskeratosis congenita [10]. Leukoplakia is most often diagnosed after the fourth decade of life and is more common in men [8].

The pathophysiology and progression of OL are likely due to a complex interplay of molecular, genetic, epigenetic, inflammatory, microbiome, immune, and other factors. MT of OL appears to possess mutations in some of the same tumor suppressor and proto‐oncogenes of Califano's genetic progression model for head and neck cancer [11, 12]. This was validated by Rosin et al. [13] where these investigators showed that allelic loss at predetermined loci increases the incidence of oral lesion progression by up to 33% as compared to those without allele loss. DNA hypermethylation has also been implicated in oral oncogenesis. Investigation has shown that increased methylation of the protein encoded by the zinc finger protein 582 (ZNF582^m^) increases the incidence of OL progression as compared to other known targets of hypermethylation [14]. Specific alterations of immune cells can be found in OL. For example, increased presence of tumor‐infiltrating lymphocytes (TILs) is found in higher grades of dysplasia [15]. Also, the presence of macrophages correlates with increased progression and transformation of OL, thought to be mediated through M2 macrophage evasion of cell death pathways [16, 17]. Dysregulation of the immune system also appears to be central to the pathophysiology of the more aggressive variant of OL, proliferative verrucous leukoplakia (PVL). Hanna et al. demonstrated enrichment of cytotoxic T cells and T regulatory cells within the subepithelial microenvironment, accompanied by overexpression of programmed death ligand (PD‐L1) compared to OL. Similarly, Fernandes et al. also demonstrated increased numbers of cytotoxic T cells within the epithelium–connective tissue interface microenvironment, different cytokine expression profiles, and an immune imbalance as measured in peripheral blood relative to OL lesions. Collectively, these findings suggest an immunologic pathogenesis of PVL [18, 19].

Of recent interest in oral oncogenesis is dysregulation of the oral microbiome as well as inflammation from periodontal disease and chronic mucosal trauma. One way in which oral dysbiosis has been thought to contribute to OL progression is by decreasing transcription of critical tumor suppressor genes [20, 21]. In regard to inflammation and the pathophysiology of OL, Goertzen et al. [22] examined oral lesions including hyperkeratosis, dysplasia and oral squamous cell carcinoma (OSCCa) and found a progressive increase in inflammatory infiltrate in lesions correlating to increasing severity of dysplasia. This inflammatory infiltrate, specifically that of neutrophil invasion, is hypothesized to increase transcription of proinflammatory cytokines that may promote lesion progression. Common sources of such inflammation include periodontal disease and chronic mucosal trauma. However, they are not demonstrably causal and require further expanded investigation [22, 23].

Investigation into the ways in which dysregulation of the oral microbiome, immune system, and inflammation promotes oral oncogenesis is in their nascent stages and requires further rigorous investigation.

The clinical appearance of OL is the basis for their classification. In their widely cited article, Warnakulasuriya et al. [1] classify OL as homogenous and nonhomogenous. They further subdivided nonhomogenous into speckled (erythroleukoplakia), nodular, and verrucous. Recently, the natural course of patients with OL has been documented in large systematic reviews which include outcomes assessment based on clinical subtype. These studies aim to quantify MT rate, which can be helpful in counseling patients regarding treatment decisions [5, 24, 25, 26]. Homogenous leukoplakia has the lowest overall lifetime MT rate (8.6%). A clinical diagnosis of erythroplakia, which is often associated with the presence of high‐grade dysplasia, confers a roughly 33% risk of lifetime MT rate [5, 25, 27]. PVL is the condition which confers the greatest yearly (9.5%) and lifetime (49.5%) risk of MT [5]. An overall pooled incidence of 9.5%–9.8% lifetime MT rate is noted when all subtypes of OL are grouped together, which is higher than previously published [5, 24, 26].

Histologic exam has long been used as an adjunct to clinical exam to aid in assessing risk of MT and thus help inform treatment management. The WHO supports a three‐tiered classification of dysplasia including mild, moderate, and severe (including carcinoma in situ) [28, 29]. A recent meta‐analysis of studies on oral epithelial dysplasia (OED) from 2015 reports a pooled MT rate of 10.5% when all degrees of dysplasia are grouped together [30]. Mehanna et al. [31] report a 10.3% MT rate for mild and moderate dysplasia and 24.1% MT rate for severe dysplasia. While other studies report lower, single digit MT rate for mild and moderate dysplasia, the consensus is that severe dysplasia confers double digit MT rate [5, 32]. Bernard, Jaber and Elameen et al. [32, 33] report mean time to MT of dysplastic OL of 3.8 and 3.3 years respectively.

While the three‐grade system remains the most used, a binary grading system (low‐risk and high‐risk) has also been proposed. Sperandio et al. [34] reported a greater prognostic value of the 3‐grade system compared to the binary system. Conversely, Freitas Silva et al. [35] suggested binary grading may be more accurate, reproducible, and predictive of MT risk than the 3‐tier system, but not sufficiently different to modify clinical decision‐making.

Nearly as common a diagnosis as oral dysplasia is atypical epithelial hyperplasia. This has also been called indeterminate dysplasia or keratosis of unknown significance due to cytological evidence of atypia without overt dysplasia. Oftentimes, these lesions present histologically with hyperorthokeratosis [36]. This histology has been reported to harbor similar genomic instability and potential for progression to malignancy as dysplasia [37]. Greater investigation into this entity is necessary to define and intervene in these lesions.

Due to a relatively high MT rate and the inability to predict lesion behavior, the clinical management of oral premalignant lesions remains a major clinical dilemma. Improvements in early detection and treatment are needed to improve outcomes. This review aims to provide an evidence‐based update for the clinical management of OL.

## 2 Clinical Management: Biopsy

Evidence‐based guidelines for the diagnosis and initial management of OL are present [38, 39, 40]. The American Dental Association's (ADA) recommendation is for short interval surveillance for seemingly innocuous lesions that are not suspicious of malignancy. It is their position that if the lesion does not resolve and the clinical diagnosis of a potentially malignant disorder cannot be ruled out, then clinicians should perform a biopsy of the lesion or refer the patient to a specialist for biopsy. For suspicious lesions or OPMDs, they recommend formal tissue biopsy or referral for one at the time of recognition [40].

Some controversy exists regarding the type and method of biopsy. A recent multispecialty survey reported that incisional biopsy is the most frequently employed biopsy method by practicing head and neck surgeons [41]. However, it is worth mentioning that when incisional biopsy is employed, sampling error could occur and lead to underdiagnosis; therefore, biopsy site selection is critical. Archibald, Buryska, and Ondrey [42] found incisional biopsy underdiagnosed dysplasia in 29% of lesions that were then subsequently excised. In fact, they report 12% of 200 incisional biopsies were subsequently identified to harbor malignancy on excisional biopsy. Underdiagnosis has been examined in other studies and found to occur most often in the setting of severe dysplasia, where excision of the lesion may lead to an upgrade in diagnosis [43]. For this reason, Archibald, Buryska, and Ondrey [42] advocate multisite biopsy or lesion excision when the clinical and histologic findings are discrepant. However, if the biopsy removes the entire lesion with primary closure of the biopsy site and high‐grade dysplasia or OSCCa is identified, there may be greater difficulty in re‐excision due to inflammatory changes and scarring. In addition, Schemel et al. [44] showed that excisional biopsies performed by practitioners who do not perform oncologic surgery may provide less pathologic information to oncologic care providers, which may increase the risk of undertreatment at the time of re‐excision, thus increasing the risk for locoregional recurrence. Other published high‐risk factors that warrant consideration for repeat of initial diagnostic biopsy or upfront lesion excision include erythroplakia, large lesional surface area, advanced age, female gender, multifocal nature, ulceration, induration and bleeding, and presence of moderate‐to‐severe dysplasia [2, 31, 45].

Exfoliative cytology has been examined as a minimally invasive adjunct to clinical exam. Currently, according to ADA guidelines, its use is only for triage of lesions when standard biopsy is unavailable [40]. However, there is current interest in incorporating optical, molecular, genomic, cytomorphometric, or machine learning into this triage technique to further aid in identifying high‐risk lesions, yet this remains investigational [46, 47, 48, 49, 50].

## 3 Clinical Management: Diagnostic Adjuncts

There are a number of diagnostic tools available that may accelerate the decision to perform and aid in biopsy site selection of OL [51]. Approaches include the use of topical agents alone or in combination with external luminescence to highlight abnormal mucosa. Toluidine blue, for example, is an acidophilic agent that, when applied to mucosal surfaces, binds to areas with higher DNA and RNA content with the goal of highlighting areas of dysplasia or malignancy. Recent systematic review and meta‐analysis by Kim et al. which included 29 studies and 1921 participants show a high pooled negative predictive value (NPV) of 71% [52]. When combined with external luminescence in this study (chemiluminescence), they report lower specificity and NPV suggesting greater accuracy with toluidine blue alone [53]. Chemiluminescence in general has been associated with low specificity which renders it an ineffective screening tool.

Tissue autofluorescence (AF) is another common screening tool that utilizes external lights alone to aid in oral lesion risk assessment. For example, Visual Enhanced Light scope (VELscope) is an instrument developed to exploit the principle that certain biofluorophores experience excitation when light at a certain wavelength is introduced. This energy is then dissipated through tissue fluorescence, which can be visualized. It has been found that diseased mucosa may result in disruption of such fluorescence, thus resulting in abnormal areas appearing darker, exhibiting loss of fluorescence as compared to surrounding mucosa. Recent systematic review and meta‐analysis of AF by Moffa et al. show a low pooled positive predictive value (PPV) of 51.3% but a higher NPV of 81.1%. Thus, if a lesion is not clinically suspicious and AF is negative, the patient may not require biopsy. However, their low PPV underlies the importance of clinical exam and assessment for need of biopsy to avoid false negatives [54]. Li et al. [55] also report higher NPV than PPV and report efficacy of AF for use in low‐risk lesions but that AF was not as accurate in identifying high‐risk lesions. Thus, interpretation of AF is dependent upon operator skill and experience and clinical exam is still critical to avoid relying on false‐negative result which can lead to missed diagnosis and treatment delay.

Another modality that has shown promise is narrow band imaging (NBI). Using this approach, a specialized light is introduced that is specific to the green and blue wavelengths (540 and 415 nm, respectively). This light, when it penetrates mucosal surfaces, is absorbed by superficial blood vessels, thus giving them a dark blue or brown color. This results in increased visibility of lesions with higher vascularity. When used in evaluation of OL, the intraepithelial papillary capillary loop classification (a scheme used to quantify the superficial vascular architecture of mucosal lesions) has been shown to be predictive of higher malignant potential. A meta‐analysis by Zhang et al. [56] including 13 studies with 1179 participants demonstrated this approach to be 87% sensitive and 83% specific when IPCL II classification or above lesions were positive on NBI assessment. While promising, this technique requires considerable expertise of the user and at present is not widely available.

There is also great interest in identifying biomarkers to predict OL lesion progression as adjuncts to clinical and histologic exam. In 2021, Monteiro, Mello, and Warnakulasuriya performed a systematic review examining the use of biomarkers for OL and found 49 candidate markers examined across 46 studies. The most frequently examined biomarkers included p53, podoplanin, and chromosomal loci abnormalities/loss of heterozygosity (LOH). In their analysis, they found significant variation in reporting and design of these studies. However, they concluded that podoplanin and chromosomal loci abnormalities have the most significant association with MT [57]. Swain et al. [58] reported a threefold increase in MT of OL that express podoplanin. The application of LOH as a biomarker to predict OL progression to malignancy has been evaluated and validated to an even greater extent than podoplanin [12, 13, 59, 60, 61, 62, 63]. DNA aneuploidy has also been suggested as a biomarker, and it is present in higher frequency with increasing grade of dysplasia. When present, it also predicts a higher rate of MT versus diploid status [64, 65].

The application of artificial intelligence/machine learning to predicting OL progression has become a topic of great interest. Examination of clinicaltrials.gov shows more active or pending trials examining machine learning algorithms for prediction of OL lesion progression than any other category of studies for OL research. Wu et al. published one of the first studies examining the use of machine learning to predict progression of premalignant lesions. They found that grade of dysplasia and presence of multiple oral lesions were most predictive of risk for transformation. In addition, they found that tongue subsite, a history of anemia, and prior history of oral cancer were also predictive covariates, though less than the aforementioned predictors [66].

In a 2017 clinical practice guideline report, the American Dental Association concluded that no available diagnostic adjuncts possess a high enough diagnostic accuracy for routine use in the diagnosis and screening of oral lesions [40]. This remains true, as all adjunctive tools require additional investigation and validation.

## 4 Clinical Management: Definitive Treatment

The first step in definitive management should involve counseling and treatment for cessation of alcohol, tobacco, betel quid, marijuana, vaping, and any other potential etiologies of OL. This can be completed as part of the initial consultation using simple cessation techniques and methods, such as nicotine replacement for tobacco smokers. Further consultations can be made for medical management of cessation, treatment of comorbid psychiatric conditions or referral to detoxification or rehab centers depending upon the need of the patient. There is a significant effort being made to bring cessation counseling to the forefront of head and neck cancer treatment, at the time of initial consultation. The same opportunity exists at the time of consultation for OL, with the potential for prevention being even greater at this earlier stage of recognition.

Treatment options for OL include observation, surgical or laser excision, laser ablation, and chemoprevention. Often, treatment depends upon the size and characteristics of the lesion. For example, a small, well‐defined, localized lesion may be amenable to excision (either surgical or laser removal) with low morbidity, whereas larger, diffuse lesions may require consideration for alternative methods such as topical or systemic chemoprevention.

In 2023, Zhou et al. completed a review of studies that examined excision of oral precancerous lesions with attention to rate of recurrence and MT. They found a pooled recurrence rate from 13 studies comprising 907 patients showed a 29.5% recurrence after scalpel excision and a 32.2% recurrence after laser excision. For patients with OL, the pooled rate of MT was 8.9% for scalpel excision, 6% for laser, and 10.2% for clinical observation, without statistically significant difference (Table 1) [67]. Thus, neither surgical nor laser excision is superior in regard to recurrence rates of OL or preventing MT. Another recent review with meta‐analysis examining laser compared to standard treatment shows no statistical difference in MT rates between scalpel and laser excision [68]. This demonstrates the need for close follow‐up regardless of treatment provided and the need for additional prospective trials to validate diagnostic adjuncts and treatment of OL to intervene in high‐risk patients.

**TABLE 1:** Pooled rates of recurrence and malignant transformation based upon OL treatment modality.

Mode of treatment	Recurrence rate (%)	Malignant transformation rate (%)
Scalpel excision	29.5	8.9
Laser excision	32.2	6
Observation	n/a	10.2

*Note*: No statistical significance exists among these methods for either category. Laser ablation is not included as little data exist regarding recurrence and MT after this more controversial method.

Laser ablation is controversial as compared to laser excision, as thoroughness of removal and margin status cannot be adequately assessed. However, some publications justify its use in certain situations. For example, large, diffuse, homogenous lesions that have been biopsied numerous times and showed no or low‐grade dysplasia may be treated with laser ablation, when excision would result in more significant morbidity [69]. Also, it might be considered in the case of high‐grade dysplasia in patients who are not surgical candidates, due to advanced age or significant medical comorbidities.

Appropriate surgical margins for excision of OL are in the range of 2–5 mm in depth and width [38, 70, 71]. Prospective evaluation of excision of OL with such margins is sparse but does exist [70, 72]. Arduino et al. examined excision vs. observation (“wait and see”) of nondysplastic OL lesions such as hyperkeratosis. In their study, they enrolled 260 patients with nondysplastic OL who were randomized to excision versus observation. One patient in each group developed oral cancer, and thus, they concluded that a “wait and see” approach is safe and with less morbidity than excision for patients with nondysplastic OL [72]. Lombardi et al. similarly examined excision vs. observation for patients with dysplastic OL. They enrolled 161 patients who were split into treatment versus observation. MT occurred in 8 instances total, of which 7 where in the observation group. Despite having a small sample size, this suggests potential efficacy for excision of dysplastic OL with 2–5 mm margins [70]. At the same time, Arduino et al. [72] trial suggests that nondysplastic OL may be observed with close clinical surveillance while avoiding morbidity of surgical treatment. Their investigation is ongoing: They have expanded their accrual and increased the duration of long‐term follow‐up to 5 years in the hopes of providing evidence‐based guidelines for management of dysplastic and nondysplastic OL.

Guidelines for management of residual dysplasia at the margin of OL excision have not been well studied. However, the presence of dysplasia at the margins after resection of early‐stage OSCCa has been examined. Sopka et al. [73] found that the presence of moderate–severe dysplasia at the margin of excision was associated with significantly worse local control (49% versus 82%) and disease‐free survival (49% vs. 80%) from OSCCa compared to specimens with only mild dysplasia or no dysplasia at the margin. Similarly, Chen et al. examined local recurrence rates of OSCCa based upon margin status of 1642 patients after oncologic resection. They found that local recurrence of OSCCa for close (21.8%) and mild/moderate dysplasia (21%) was similar as compared to clear margins, which was lower (15%) [74]. It is unclear whether the presence of mild or moderate dysplasia at the margin of OL excision incurs this same potential increase risk of recurrence and this warrants further study.

## 5 Chemoprevention and Field Cancerization

Several decades worth of research has been carried out on oral cancer chemoprevention. This work has been summarized in two recent publications. A Cochrane review in 2016 examined all previous oral cancer chemoprevention literature which included many of the landmark trials commonly referenced such as retinoids, COX inhibitors, antioxidants, and other supplements. The conclusion was that no single agent demonstrated durable efficacy without side effect. Therefore, all interventions were recommended for continued investigation [75].

In 2024, a similar review was conducted. It had some overlap with the Cochrane analysis in discussing historical trials such as those investigating Vitamin A and the retinoids, lycopene, celecoxib, ketorolac, bleomycin, green tea extract, and dried black raspberry gel. However, it included several new classes and types of medications being investigated such as EGF inhibitors, metformin, and immunotherapy [76].

Gutkind et al. investigated metformin as a chemoprevention agent for OL given its presumed activity against mTOR/PI3K pathways which are implicated in OSCCa. They identified 17% clinical response and 60% at least partial histologic response after a 12‐week course of metformin (*n* = 23). Furthermore, they found that decreased mTOR activity in the basal epithelial layer of OL correlated to clinical (*p* = 0.04) and histologic (*p* = 0.01) response [77]. These data formed the justification to proceed with a currently ongoing phase IIB study (NCT05237960). Additionally, there are several other clinical trials investigating metformin and pioglitazone either alone or in combination for the treatment OL (NCT00099021, NCT00951379, NCT05727761, NCT05727761).

Hanna et al. conducted a phase II nonrandomized controlled trial (NCT03692325) to assess the efficacy of nivolumab in PVL. Patients (*n* = 33) were given four cycles of nivolumab (28 day cycles). Overall, patients were able to tolerate therapy (88% completed treatment). Nine patients demonstrated a partial response (40%–80% decrease in dysplasia composite score), three had a major response (> 80% decrease in dysplasia composite score), 16 had stable disease, and four had progression of disease over a median follow‐up of 21.1 months. They observed a 2‐year cancer‐free survival in this cohort of 73% (27% patients developed OSCCa during the trial: six had previous history of OSCCa, and three had shown treatment response). On whole‐exome analysis of specimens, the authors noted potential correlation of 9p21.13 loss and progression to OSCCa (6/6 patients with OSCCa had the deletion, while 4/14 of patients with no invasive cancer had the deletion) [78].

A multicenter study of the oral anti‐EGFR medication erlotinib was conducted by William et al. in aims of preventing oral cancer. Although their use of erlotinib in the targeted treatment for OL was not found to improve 3‐year cancer‐free survival relative to placebo, they successfully validated the use of LOH for stratifying patients into low‐ and high‐risk groups. Low‐risk patients were found to have an 86% cancer‐free survival at 3 years compared to 74% in high‐risk patients. This first prospective validation of LOH presents a promising ongoing research avenue to risk‐stratify patients and to provide a molecular adjunct to clinical and histologic diagnosis [63]. In addition, it prospectively validates the genetic progression models and LOH research by Califano et al., Rosin et al., and Zhang et al. [12, 13, 62].

Other potentially promising trials using topical agents for oral cancer chemoprevention include imiquimod and photodynamic therapy (PDT). Recently, Sroussi et al. investigated the use of topical imiquimod for the treatment of OL. This immunomodulator activates Toll‐like receptor 7 and has been found to be previously efficacious in treating certain skin lesions. In a cohort of 33 patients, they noted 68.4% of OL lesions were reduced in size by over 50%, and 42.1% demonstrated complete resolution [79]. Additional trials investigating the efficacy of topical application of imiquimod for OL show similar promise, even in the difficult subset PVL, and this requires further investigation and research [80, 81, 82].

Photodynamic therapy of OL has been assessed using a number of photosensitizers including 5‐Aminolevulinic acid, toluidine blue, methylene blue, and others with evidence of promising effects [83]. A prospective case series of 11 patients with 15 lesions treated with photodynamic therapy and topical toluidine blue as a photosensitizer reported complete response in approximately one‐third of treated lesions at the completion of local therapy and partial response in half of lesions with up to one‐year follow‐up posttreatment [84]. However, there are several disadvantages: Lesions do recur, application of photodynamic therapy is cumbersome and time‐consuming, and studies only track lesion response in the short term, without evaluating for long‐term risk of developing OSCCa [85, 86, 87]. These studies suggest potential topical approaches for the management for localized oral premalignant lesions.

These trials represent an important paradigm shift from observational approaches to medical interventions in at‐risk patients. While these have not been fully validated for clinical standard of care, further investigation and stratification of patients based upon risk will bolster scientific evidence to promote early interventions in these patients.

## 6 Prognosis/Long‐Term Management

All white and red lesions of the mouth have some risk of MT [5]. Most cancers are detected within 3–5 years of diagnosis of a premalignant lesion; however, the risk of MT continues for at least 10–15 years [3]. A recent randomized trial shows efficacy of clinical surveillance every 6 months for white nondysplastic lesions as compared to surgical excision, with equivalent transformation rates in the two groups, and mean time to conversion of 49.5 months. They also justify that 6‐month surveillance is a reasonable interval as de novo lesions would not likely develop into a cancer in less time [72]. There are no prospective, consensus guidelines for the surveillance of patients with dysplasia. However, Archibald, Buryska, and Ondrey outlined an active surveillance protocol based upon clinical and histologic features that included length and frequency of follow‐up, as well as timing of repeat biopsy. For mild dysplasia or hyperkeratosis, patients returned every 6–12 months; for moderate and severe dysplasia, they returned every 3 months for exam. Furthermore, they advocated for re‐biopsy of all conditions with the following minimal frequency: at least every 2 years for mild dysplasia, 12–18 months for moderate dysplasia, and 3–9 months for severe dysplasia unless there is obvious clinical progression. For all these conditions, they recommend active surveillance for at least 5 years of duration [42]. These guidelines provide a framework for patient counseling and clinical management, though ultimately joint patient–doctor decision‐making supercedes any guidelines should there be concern for, or lack of progression. However, this article highlights the importance of clinicopathologic correlation and repeat biopsy is an important part of active surveillance of oral preneoplasia.

## 7 Conclusion

The identification of high‐risk OL represents a diagnostic dilemma. Several candidate biomarkers exist and require additional study for validation. Clinical management of leukoplakia depends on the size, location, and clinical and histologic features of the lesion. Limited prospective data show nondysplastic OL can be managed with close clinical surveillance. For lesions found to be dysplastic, formal excision or ablation may decrease MT rates and should be employed assuming no contraindication. Neither laser nor scalpel excision has been proven superior over another in preventing malignant transformation. Many nonsurgical treatments are the subject of ongoing clinical trials and may serve to aid those with diffuse or recurrent lesions, where surgery presents unacceptable morbidity. In all instances, close clinical surveillance is paramount to monitor for lesion recurrence, disease progression, and malignant transformation. Further prospective, randomized study is needed to better understand the pathogenesis of this disease and to better inform treatment decisions.

## Data Availability

Data sharing is not applicable to this article as no new data were created or analyzed in this study.
